# Experimental and Computational Approaches of Newly Polymorphic Supramolecular *H*-Bonded Liquid Crystal Complexes

**DOI:** 10.3389/fchem.2020.571120

**Published:** 2020-11-02

**Authors:** Laila A. Al-Mutabagani, Latifah Abdullah Alshabanah, Magdi M. Naoum, Mohamed Hagar, Hoda A. Ahmed

**Affiliations:** ^1^Chemistry Department, College of Science, Princess Nourah Bint Abdulrahman University, Riyadh, Saudi Arabia; ^2^Department of Chemistry, Faculty of Science, Cairo University, Cairo, Egypt; ^3^Chemistry Department, College of Sciences, Taibah University, Yanbu, Saudi Arabia; ^4^Chemistry Department, Faculty of Science, Alexandria University, Alexandria, Egypt

**Keywords:** 4-(nicotinoyloxy) phenyl nicotinate, supramolecular H-bonded complexes, optical materials, computational calculations, DFT

## Abstract

New 1:2 liquid crystalline supramolecular H-bonded complexes (SMHBCs) were synthesized through double H-bond interactions between 4-(nicotinoyloxy) phenyl nicotinate as the base component and two molecules of 4-n-alkoxybenzoic acid (An). The base component was expected to be in two conformers according to the orientation of the N atom and the carboxylate group, *syn* conformer (**I**) and *anti*-conformer (**II**). DFT calculations revealed that only one of the two possible conformers of **I** exists, and the addition of the two molecules of the alkoxy acids (**A***n*) did not affect its conformation. The mesomorphic properties of all of the prepared complexes (**I**/**A***n*), bearing different terminal flexible alkoxy chains were investigated, and the formation of the H-bonds were confirmed by differential scanning calorimetry (DSC), and the phases were identified by polarized optical microscopy (POM), and FT-IR spectroscopy. Highly thermally stable mesophases possessing broad temperature ranges were observed for all investigated complexes compared to their individual components. Depending on the length of the terminal flexible alkoxy chain, the prepared SMHBCs were shown to exhibit di- or tri-morphic enantiotropic mesophases. The effect of replacing one of the –COO– connecting units by an azo group (-N=N-) in the basic molecule (**I**), on the mesomorphic properties has been investigated experimentally (*via* DSC) and theoretically (*via* DFT). The DFT calculations revealed that the polarizability, the dipole moment, and the aspect ratio of the investigated SMHBCs are lower than those of their corresponding ester/azo analogs. All these factors rationalize the enhanced smectic mesophase ranges of the complexes compared with those of the ester/azo analogs. The high aspect ratios and dipole moments of the SMHBCs of the azo derivative enforces the lateral intermolecular attraction that permits the formation of the more ordered smectic C mesophase with respect to the enhanced polymorphic mesophases of the diester derivative.

## Introduction

Liquid crystalline materials (LC) are compounds that exhibit one or more metaphases that are observed between the solid and the isotropic phases. LCs are used in optical imaging industrial applications (Kraft et al., 2000). On the other hand, thermotropic LCs based on intermolecular *H*-bond interactions are mostly applied in display devices and sensor applications (GonzáLez-RodríGuez and Schenning, 2011; Yan et al., 2012; Liu et al., 2013; Yang and Urban, 2013; Dong et al., 2015). The *H*-bonding history of LCs started in the twentieth century (Fairhurst et al., 1998). The first reported *H*-bonded dimers were made between 4-n-alkoxybenzoic acids and 4-n-alkoxycinnamic acids that showed stable nematic (N) and smectic mesophases (Bennet and Jones, 1939; Kato and Frechet, 1989; Kato, 2000). Molecular geometry in liquid crystalline H-bonded complexes can be assembled between different conformations dependent on the investigated *H*-bond donors and acceptors. Further, the phase behavior/molecular structure relationships are helpful tools used to prepare new materials possessing the desired properties for instrumental applications (Imrie et al., 2009; Yeap et al., 2009, 2011, 2015). The design of new materials possessing novel architectures represents an important area of our interests in geometrical approaches (Yagai and Kitamura, 2008; Yeap et al., 2009; Hagar et al., 2019). Recently, many researchers (Paleos and Tsiourvas, 2001; Armstrong and Buggy, 2005; Tschierske, 2013; Goodby et al., 2014; Ahmed and Naoum, 2016; Ahmed et al., 2018a,b; Ahmed et al., 2019b; Saccone et al., 2019; Tuchband et al., 2019; Martinez-Felipe et al., 2020; Walker et al., 2020) have investigated the mesophase behavior of new SMHBCs between the carboxylic acid group and pyridine-containing compounds (Al-Mutabagani et al., 2020; Nafee et al., 2020). These studies were focused on the correlation between the mesomorphic transition data and the evaluated computational calculations for SMHBCs. Kato et al. reported the investigation of 2:1 supramolecular *H*-bonding interactions between two dissimilar mesogens, namely, trans-p-[(pethoxybenzoyl]oxy]-p′-stilbazole and 4-alkoxybenzoic acids (Kato et al., 1992, 2006; Kato, 2000; Yagai and Kitamura, 2008; Jeong, 2011). Another type of H-bonding assembly is the bent (angular) shaped (-shaped) complexes, as that reported for the 1:2 phthalic acid and stilbazole derivative interactions (Kato et al., 1992). These two individual components are non-mesomorphic, whereas, their H-bonded assembly showed induced nematic mesophases (Kato et al., 1992). Geometrical conformations of these SMHBCs depend on their molecular shape that have an important role in the enhancement of the mesophase stability.

Recently, it was observed that the possible orientation of hetero-atoms in pyridines is used to modify the existing functions thus introduces a new geometrical characteristic to the organic molecules (Al-Mutabagani et al., 2020; Nafee et al., 2020). Further, the mesogenic core, terminal alkoxy chains, and terminal polar substituents were shown to play important roles in the formation, type, thermal stability, and temperature range of the LC compound mesophases. In this case, molecules tend orient themselves in parallel alignments as the length of the terminal substituent increases (Dave and Menon, 2000). In addition, the terminal alkyl or alkoxy chains will influence the nematic twist bend and heliconical phases (Abberley et al., 2018; Paterson et al., 2018).

Going further in our investigation concerning SMHBCs, new five ring architectures of 1:2 supramolecular *H*-bonded complexes, based on symmetrical di-nicotinate base moiety (**I**) and 4-n-alkoxybenzoic acids (**A***n*), as H-donors, were prepared.

The aim of the present work is to investigate the mesomorphic, optical properties, and geometrical parameters of newly prepared supramolecular complexes (**I/A***n*). The study is extended to investigate theoretical calculations (DFT) and experimental measurements to show how these variables are affected by the different orientations of the two nitrogen atoms included within the predicted conformers. Finally, it is to study the effect of the exchange one of the –COO– connecting units of the present di-ester complexes with our previously reported SMHBCs of their azo analog (-N=N–), on the mesomorphic properties and other thermal parameters.

## Methods

### Synthesis of 4-(Nicotinoyloxy)Phenyl Nicotinate (I & II)

*N, N*′-dicyclohexylcarbodiimide (DCC, 0.02 mol) and 4–dimethylaminopyridine (DMAP) (as a catalyst) were added to a solution and nicotinic acid (1.23 g, 10 mmol) in 25 ml dry methylene chloride (DCM, 25 ml). The reaction mixture was kept at room temperature with stirring for 72 h. The separated byproduct was filtered off and the product was separated from the filtrate by evaporation. The solid product that remained was purified by crystallization twice from pure ethanol.

Yield: 95.0 %; FTIR ($\overset{´}{\upsilon }$, cm^−1^): 2920-2855 (CH_2_ stretching), 1743 (C=O), 1618 (C=N),1601 (C=C), 1470 (C–O_Asym_), 1242 (C-O _Sym_). ^1^H NMR (400 MHz, CDCl_3_) δ ^1^H NMR (850 MHz, CDCl_3_) δ 9.44 (s, 2H), 8.91 (dd, *J* = 4.9, 1.7 Hz, 2H), 8.51 (dd, *J* = 7.9, 1.9 Hz, 2H), 7.53 (ddd, *J* = 7.8, 4.9, 0.7 Hz, 2H), 7.36 (s, 4H). ^13^C NMR (214 MHz, CDCl_3_) δ 163.73, 154.05, 151.31, 148.19, 137.85, 125.44, 123.63, 122.73.

### Preparation of the Complexes I/A*n*

**Scheme 1:**
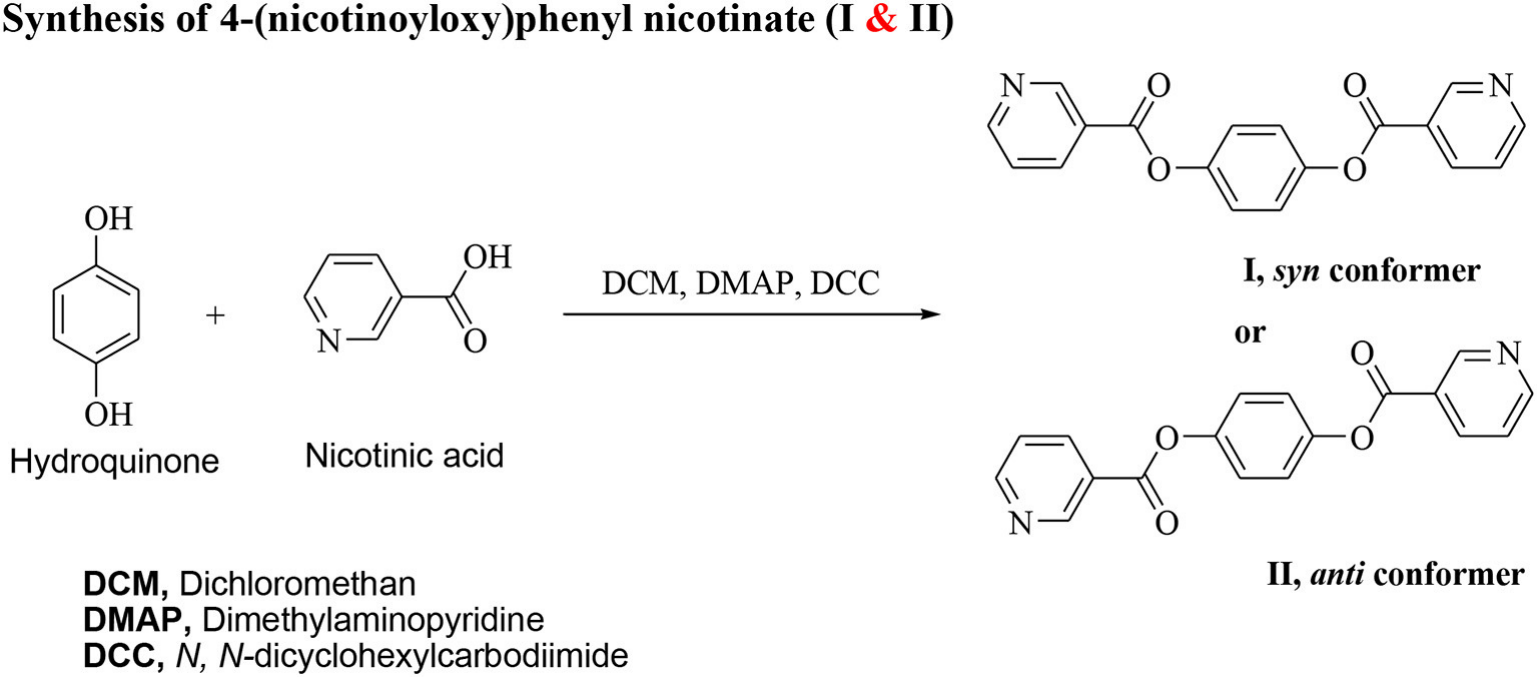
Both conformers of di-nicotinate base **I, II**.

The predicted stable supramolecular complexes **I/A***n* ware prepared from 2:1 molar ratios of alkoxy benzoic acids (**A***n*), where *n* = 6–16, and the expected stable *syn* conformer of di-nicotinate base (**I**, Scheme 1), respectively. This was done by melting the mixture with stirring to give an intimate blend, then allowed to cool. The formation of supramolecular complexes (**I/A***n*) were proved *via* DSC and FT-IR spectroscopy. DSC measurements were recorded on small samples (2–3 mg) enclosed in aluminum pans. The DSC heating rate was 10°C/min in nitrogen gas (30 ml/min) as an inert atmosphere and all transitions reported were collected from the second heating scan.

## Results and Discussion

### Supramolecular H-Bonded Complexes I/A*n* and II/A*n*

The non-mesomorphic nitrogen based component was prepared by the esterification of hydroquinone with two molecules of nicotinic acid. The diester base was estimated to exist in two different conformers depending on the orientation of the two carboxylate groups (COO), namely, the *syn* conformer **I** and *anti*-conformer **II**.

**Scheme 2:**
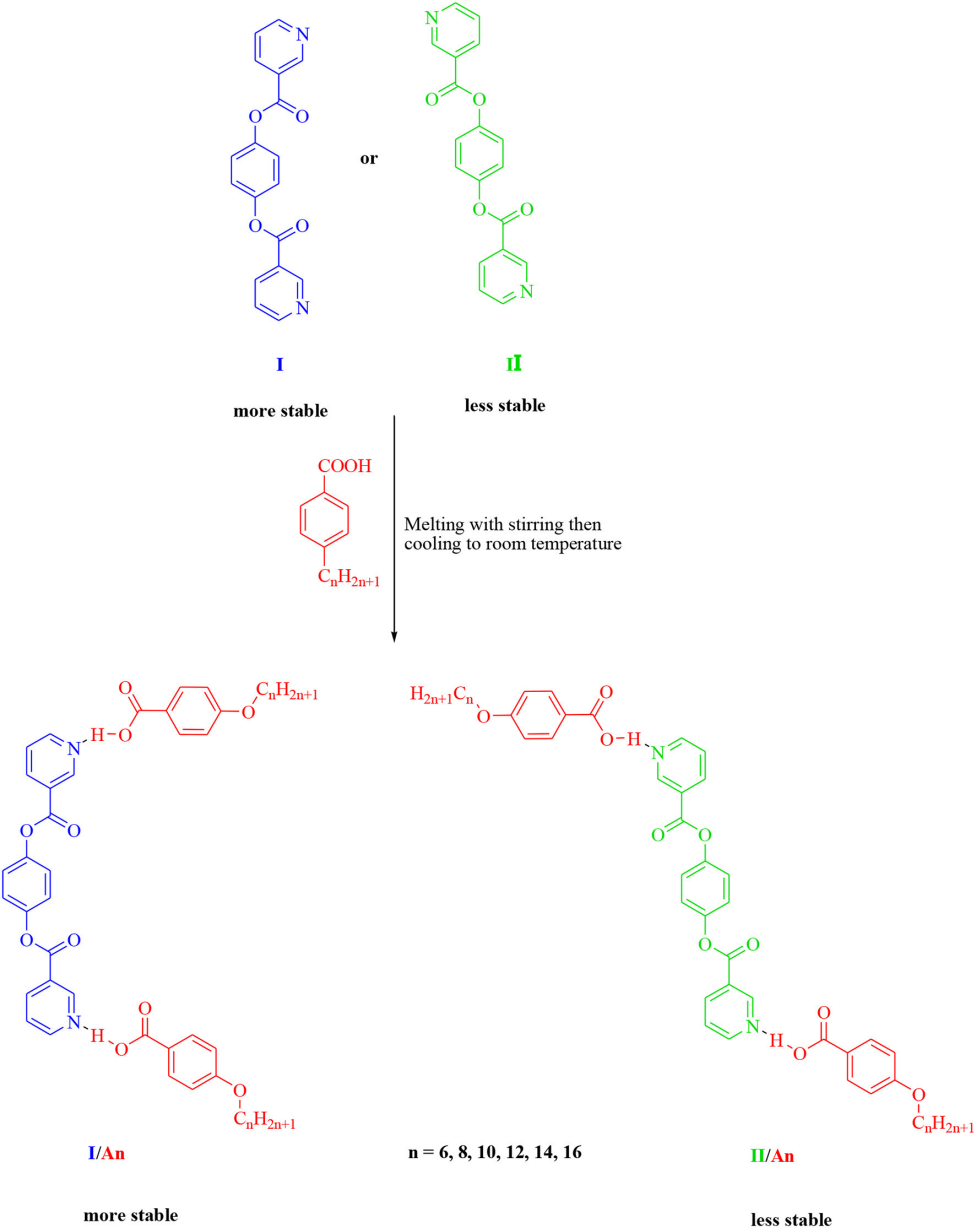
Formation of 2:1 supramolecular *H*-bonded complexes (**I/A***n* and **II/A***n*).

The 2:1 molar ratios of the alkoxy benzoic acids (**A***n*) (chain length from *n* = 6–16) and diester base (**I** and **II**) were used for the formation of the supramolecular complexes **I/A***n* and **II/A***n*, Scheme 2. As will be discussed later on, the DFT calculations revealed that only one, *syn* conformer **I**, of the two possible conformers of the diester base exists, and the addition of the two molecules of the alkoxy acids (**A***n*) did not affect its conformation.

### FT-IR Characterizations

**Figure 1:**
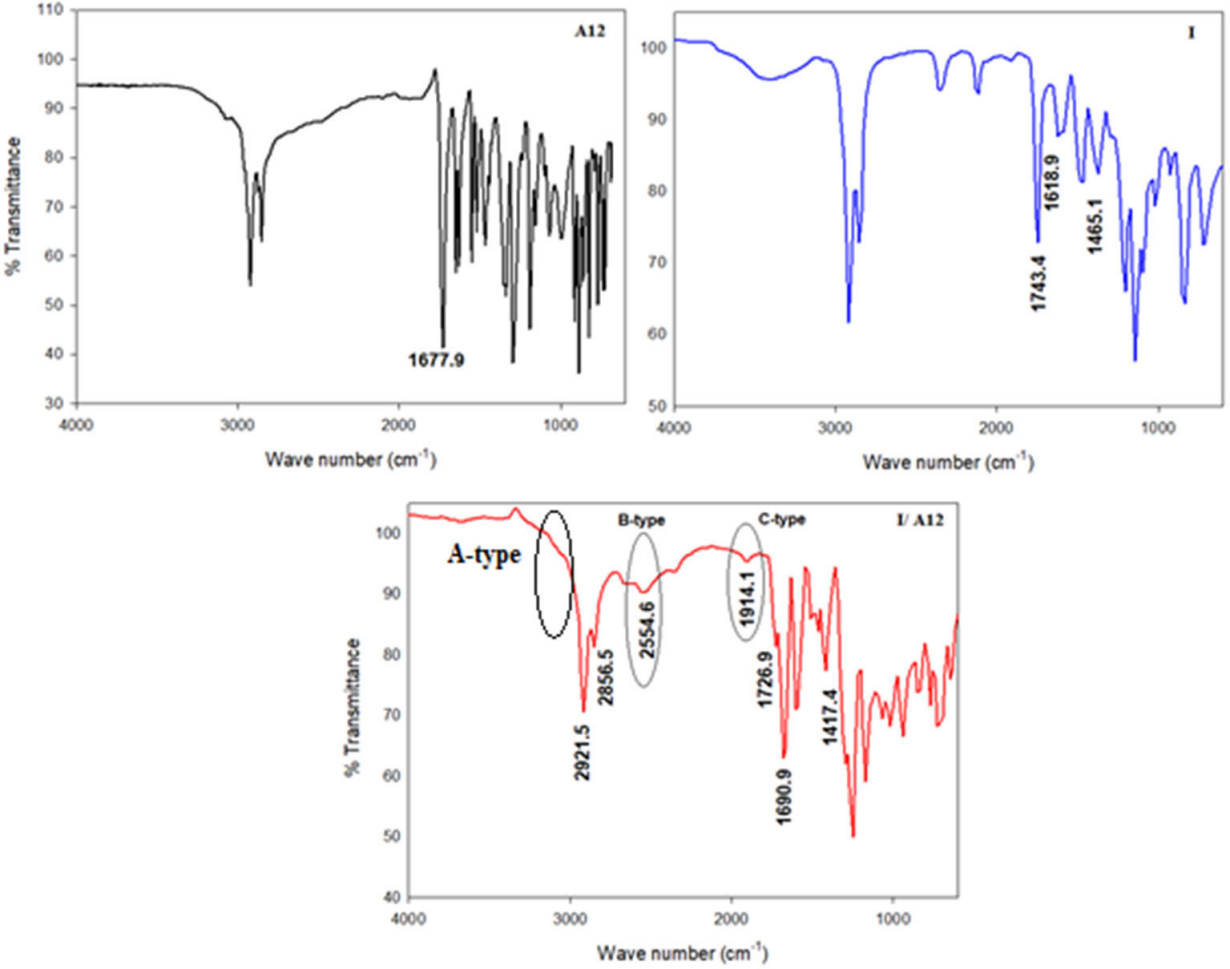
FT-IR spectra of **I**, **A***12*, and **I/A***12*.

The formation of the supramolecular complex of the di-nicotinate base **I** and the alkoxy acid **A***n* could have been proven *via* FT-IR, x-ray, or NMR spectral analysis (Saunders and Hyne, 1958; Lam et al., 2016; Martinez-Felipe et al., 2016; Hu et al., 2017; Pothoczki et al., 2020). FT-IR measurement has proven to be an effective tool for such confirmation and so this is what was used (Martínez-Felipe and Imrie, 2015; Paterson et al., 2015; Martinez-Felipe et al., 2016; Alhaddad et al., 2020; Nafee et al., 2020). The measurements of the spectral data were recorded for both of the individual components, as well as for their supramolecular complexes. The FT-IR spectra of the individual components, **I** and **A12**, and their complex, **I/A12**, were measured and given in Figure 1.

As shown from Figure 1, the signal at 1,677 cm^−1^ is assigned to the ester C=O group of the alkoxy benzoic acid **A***n*. On the other hand, the ester C=O group of the base **I** appeared at 1,743 cm^−1^. The replacement of the dimeric *H*-bond between the alkoxy acid **A***n* with that between it and the nitrogen atom of the di-nicotinate base (**I**) affects the strength of the C=O bond of either the acid or the base, and consequently, their stretching vibrational frequencies will be changed. This information could be confirmed by the FT-IR measurements. The new *H*-bonding decreases the C=O stretching vibration of the ester linkage of the base **I** to 1726.9 cm^−1^, while it increases that of the C=O of the COOH group of the alkoxy acid **A***n* to 1,790 cm^−1^.

One of the important reported ways of confirming SMHBCs complex formation is OH Fermi vibrational stretching bands (Ghanem and Noel, 1987; Cleland and Kreevoy, 1994; Lizu et al., 2010; Martínez-Felipe and Imrie, 2015; Paterson et al., 2015; Martinez-Felipe et al., 2016; Walker et al., 2018). It has been reported that the appearance of three Fermi resonance vibration bands due to the *H*-bonded OH groups, these are of the **A**-, **B**-, and **C**-types, are evidence for the formation of the supramolecular complex. The **A**-type Fermi band of complex **I/A***12* lay under that of the C-H vibrational peaks at 2,921 to 2,856 cm^−1^. Whereas, the peak at 2,554 cm^−1^ (**I/A***12*) is assigned to the **B**-type of the in-plane bending vibration due to the O-H group. On the other hand, the band at 1,914 cm^−1^ corresponds to the **C**-type Fermi band assigned to the interaction between the torsion overtone effect and the stretching vibration of the OH group.

### Mesomorphic and Optical Activities

**Figure 2:**
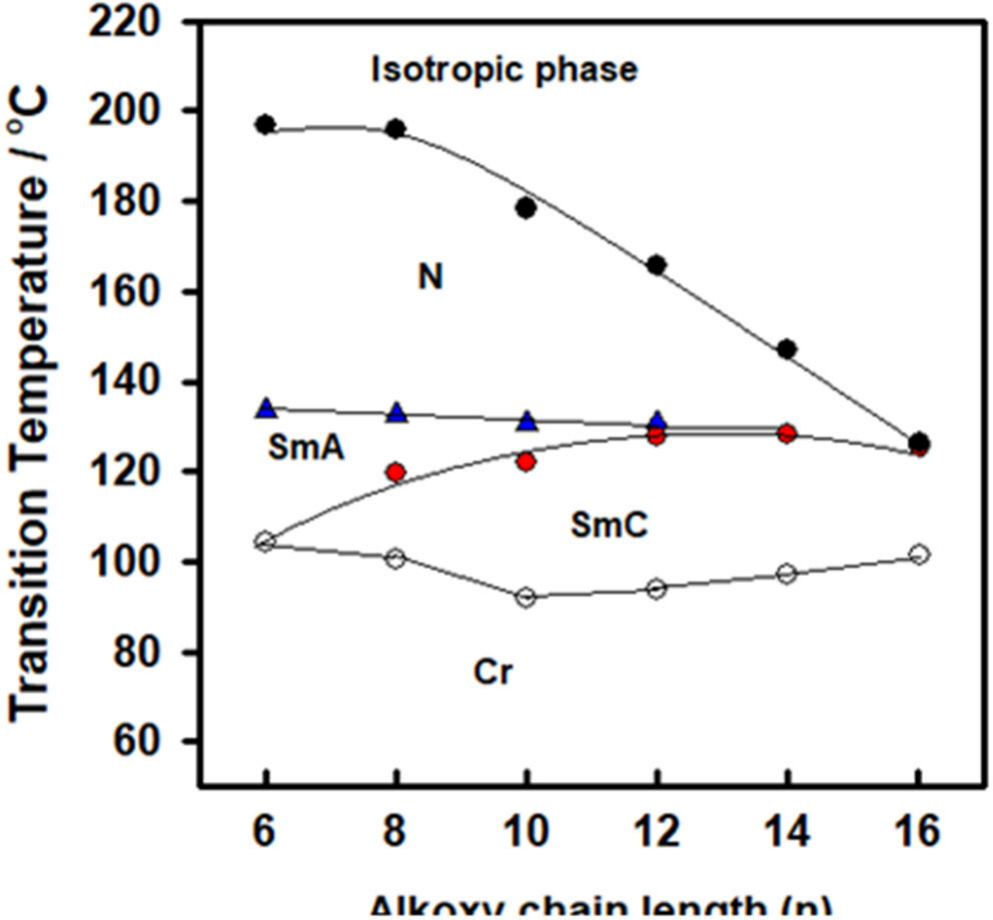
Graphical DSC transitions of 1:2 supramolecular complexes series, **I/A***n*.

The mesophase and optical analysis for the five ring 1:2 symmetrical SMHBCs (**I/A***n*) were investigated. Transition temperatures (***T***), their associated enthalpy (**Δ*H***), and their normalized entropy (**Δ*S/R***) of all mesophase transitions, as derived from DSC measurements, for all prepared SMHBC complexes are collected in Table 1. Figure 2 shows the graphical representation of the chain-length/transition temperature dependences of the investigated complexes, **I/A***n*, in order to evaluate the effect of the length of terminal flexible chains of the acid component on the mesomorphic properties.

**Table 1:** Phase transition temperatures (°C), enthalpy of transitions (**ΔH**, kJ/mol), and normalized entropy of transition (**Δ*S***/R) for the supramolecular complexes **I/A***n*.

**System**	***M.wt***	***T*_**Cr-C**_**	**Δ*H*_**Cr-C**_**	***T*_**Cr-A**_**	**Δ*H*_**Cr-A**_**	***T*_**C-A**_**	**Δ*H*_**C-A**_**	***T*_**A-N**_**	**Δ*H*_**A-N**_**	***T*_**C-N**_**	**Δ*H*_**C-N**_**	***T*_**N-I**_**	**Δ*H*_**N-I**_**	**Δ*S/R***
**I/A6**	764.8	-	-	104.1	77.85			133.6	6.69	-	-	196.7	4.64	1.19
**I/A8**	820.9	100.4	78.80	-	-	119.8	3.96	132.7	8.13	-	-	195.9	3.96	1.02
**I/A10**	876.9	91.7	71.52	-	-	121.90	2.92	130.8	7.54	-	-	178.4	3.18	0.85
**I/A12**	932.9	93.6	75.27	-	-	127.60	6.65	130.9	8.26	-	-	165.6	3.64	1.00
**I/A14**	988.9	97.0	71.86	-	-	-	-	-	-	128.2	7.35	147.2	3.00	0.86
**I/A16**	1044.9	101.2	78.92	-	-	-	-	-	-	125.5	7.54	126.5	4.55	1.37

*M.wt, molecular weight (g/mol); Cr-C, transition from solid to the SmC mesophase; Cr-A, transition from solid to the SmA mesophase; C-A, transition from SmC to the SmA mesophase; A-N, transition from SmA to the N mesophase; C-N, transition from SmC to the Nematic mesophase; N-I, transition from Nematic to the isotropic phase*.

**Figure 3:**
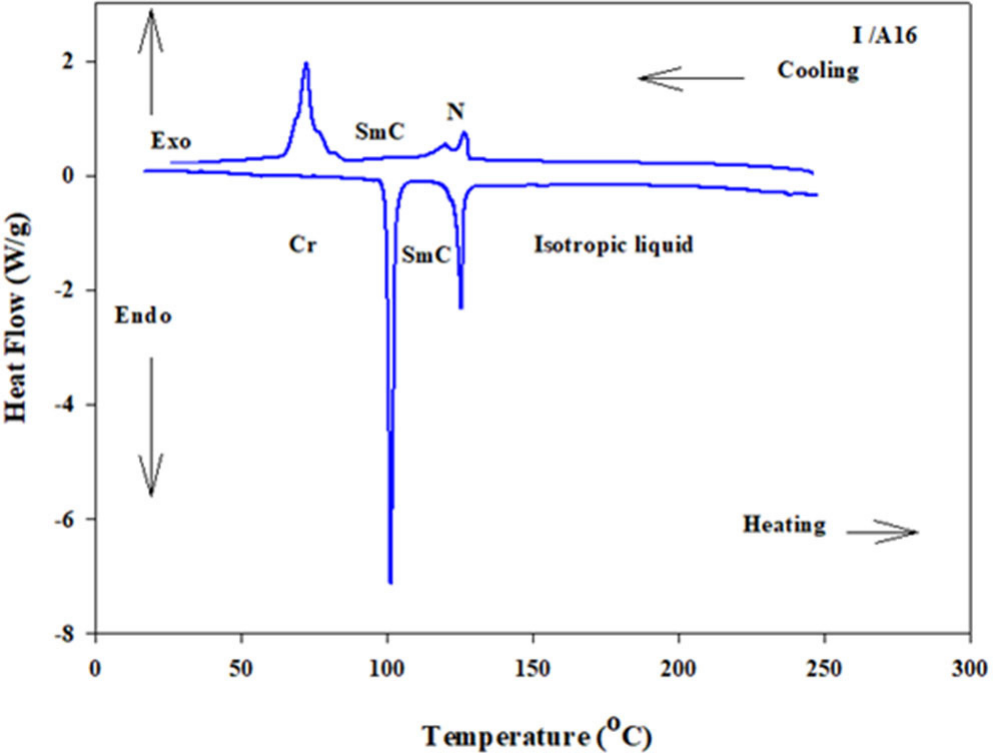
DSC thermograms upon the second heating/cooling cycles of 1:2 SMHBC **I/A***16* with a heating rate of 10 °C/min.

**Figure 4:**
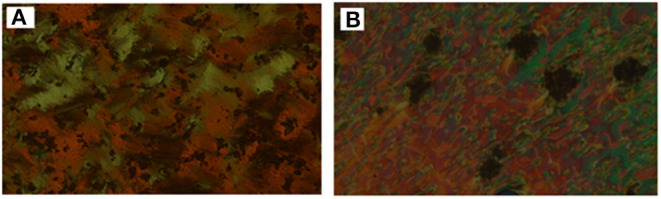
POM images for SMHBC **I/A***16* **(A)** SmC phase at 102.0°C on heating; **(B)** N phase at 126.0°C on cooling.

Analyses of Table 1 and Figure 2 indicate that, enantiotropic mesophases are exhibited by all of the prepared complexes. On the other hand, the N phase stability ***(T***_***N*−*I***_) and its temperature range ***(*Δ*T***_***N***_) are decreasing gradually with the increment of the length of the acid alkoxy-chain (see Figure 2). Conversely, the melting temperatures of the supramolecular *H*-bonded complexes showed irregular trends. The higher melting temperature shown by the **I/A***6* supramolecular complex reflects the greater co-linearity of this complex which results in more efficient packing in the solid crystal phase, in addition to the enhanced molecular interactions between the ester linkages. The stabilities of the smectic A and smectic C are also both affected by the increase of the acid alkoxy chain length (*n*) (Naoum et al., 2010) The optical properties of the diester base **I** show that it is non-mesomorphic; while, the 4-n-alkoxy benzoic acids, **A***n*, is dimorphic possessing the smectic C (SmC) and the nematic (N) phases depending on their alkoxy-chain length (Naoum et al., 2010). Although the diester component **I** does not exhibit any mesophases, all its formed 1:2 supramolecular mixtures **I/A***n* exhibit induced broad smectic and nematic mesophases with relatively higher thermal stabilities. Thus, for the shortest complex **I/A***6*, it is enantiotropically dimorphic possessing both SmA and N phases with high thermal stability (196.7°C). For the complexes, **I/A***8*, **I/A***10*, and **I/A***12*, tri-phases are observed, namely, SmC, SmA, and N mesophases. For longer acid chains (*n* = 14 and 16) complexes **I/A***14* and **I/A***16* exhibit only dimorphic phases, the SmC, and narrow N phase range. In conclusion, the lengths of the terminal alkoxy chains on the acid moiety are more effect on the stability of the formed mesophases. The SmA mesophase starts from *n* = 6 and vanished at *n* =12, while the SmC starts from *n* = 8 to *n* = 16 and become predominant from *n* = 14. The nematic phase covers all chain lengths but declines upon increasing *n* as a result of the dilution of the rigid mesogenic core (Imrie and Taylor, 1989; Imrie, 2006). That is, as the length of terminals increases, the rigidity of the central core of the supramolecular H-bonded complex decreases, and accordingly, the linearity of the SMHBCs slightly decreases as a result of the greater number of configurations of the chains that lead to the strong terminal interactions (Ahmed et al., 2019a). Representative DSC thermograms taken from the second heating/cooling scans for the supramolecular complex **I/A*16*** are shown in Figure 3. POM investigation*s* confirm the DSC results (Figure 4).

Normalized entropy of the nematic-to-isotropic transition (**Δ*S/R***) were calculated, formed DSC thermograms, for all investigated SMHBCs, **I/A***n*, and the data are collected in Table 1. Results indicate that the variation in the entropy changes when the terminal flexible-chain length varies in an irregular manner. This may be attributed to differences in the molecular interactions between individual molecules. These were found to be the differences in polarizability, dipole moment, aspect ratio, and their geometrical structures (Lizu et al., 2010).

The different order and different self-assembly within the molecular arrangement occurring for different observed mesophases contribute to the significant modification of the neighborhood packing. Moreover, the strong anisotropic dispersion potential between the aromatic rings enhances the anti-parallel orientational order. The alterations of these orientational orders within different mesophases impact the system to affect the intermolecular attractions and hydrogen bonding interactions according to previous findings (Chakraborty et al., 2018).

### DFT Theoretical Calculations

#### Molecular Geometry of SMHBCs

**Figure 5:**
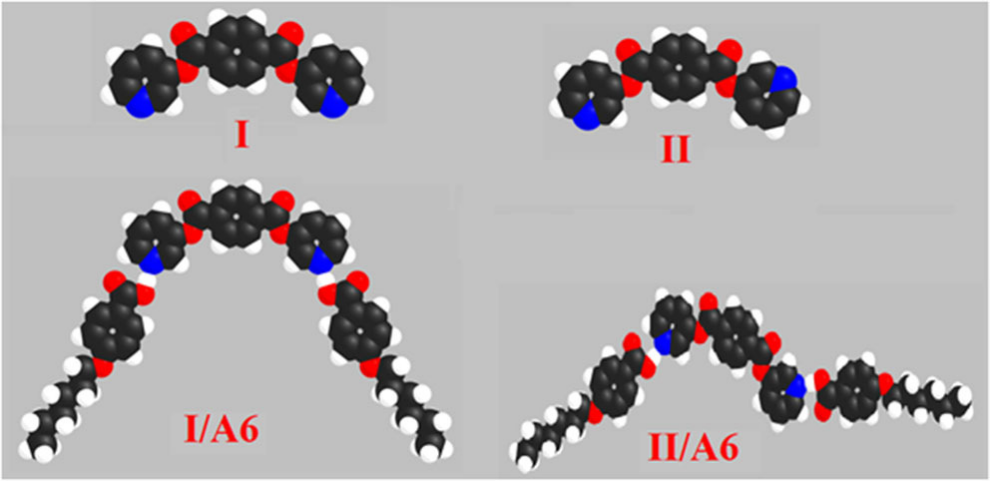
Calculated molecular geometrical conformers of the base **I**, and their SMHBCs, **I/A***6*, and **II/A***6*.

The prepared diester base is assumed to exist in two conformers, **I** (*syn*), **II** (*anti*). These conformer structures are based on the directions of the two nitrogen atoms and the carboxylate group. Moreover, the SMHBCs of the proposed conformers of the di-nicotinate are estimated to be in two conformers (**I** and **II**). The calculation of the structural and thermal parameters is made to study the effect of isomerism on the predicted and experimental findings. The theoretical calculations were performed by the DFT method at B3LYP 6-31G (d,p) for all possible conformers of the base (**I, II**) as well as its supramolecular *H*-bonded complexes **I/A***n* and **II/A***6*. The absence of imaginary frequencies can be taken as evidence for their geometrical stability. It is worth mentioning here that although the present calculations provide us with the preferred molecular geometry in the gaseous state, the presence of the liquid crystalline compounds in the condensed mesophases, the lowest energy may be different and the more elongated species are preferred (Paterson et al., 2017). Figure 5 shows the most favorable geometries for the two conformers of the base (**I**, **II**) and their *H*-bonded complexes with 4-hexyloxybenzoic acid (**I/A***6*, **II/A***6*).

Although both conformers are in their planar geometry, their derived 4-alkoxybenzoic acids SMHBCs are nonlinear. Figure 5 emphasizes that the direction of the *N*-atoms of the di-nicotinate base **I** highly affects their structural geometries. The mesomorphic behavior of liquid crystal complexes is found to be strongly dependent on the length of the alkoxy wings. This is accounted for in terms of their molecular shape (Hagar et al., 2019).

#### Thermal Parameters

The predicted thermal parameters were estimated (by DFT) applying the same method at the same set for both estimated conformers of the di-nicotinate base and their prepared *H*-bonded complexes (**I/A***n* and **II/A***6***)**, the results are tabulated in Table 2. The results of the calculated thermal parameters revealed that the *syn* conformer **I** of the di-nicotinate base is more stable than that of its geometrical *anti* isomer **II**. The extra stability of **I** could be explained in terms of the conjugation point of view, moreover, the presence of the nitrogen atoms in one side of the di-nicotinate base could permit a high degree of packing rather than the other. To check if the *H*-bonded complexation affects the stability, **I/A***6* and its constitutional isomer **II/A***6* thermal parameters are calculated for the same alkoxy chain length of the acid (*n* = 6). The results showed that the conformer **I/A***6* is more stable than its isomer **II/A***6*.

**Table 2:** Thermal parameters (Hartree/Particle) of both conformers of the *H*-bonded complexes **I/A***n* and **II/A***6*.

**Parameters**	**I**	**II**	**I/A6**	**I/A8**	**I/A10**	**I/A12**	**I/A14**	**I/A16**	**II/A6**
E_corr_	0.269	0.269	0.856	0.971	1.086	1.201	1.316	1.431	0.856
ZPVE	−1102.953	−1102.952	−2565.853	−2722.954	−2880.056	−3037.157	−3194.258	−3351.359	−2565.852
E*_*tot*_*	−1102.934	−1102.932	−2565.798	−2722.893	−2879.989	−3037.085	−3194.181	−3351.277	−2565.797
H	−1102.933	−1102.932	−2565.797	−2722.892	−2879.988	−3037.084	−3194.180	−3351.276	−2565.796
G	−1103.006	−1103.005	−2565.965	−2723.073	−2880.182	−3037.291	−3194.399	−3351.509	−2565.960

*ZPVE, Sum of electronic and zero-point energies; E_tot_, Sum of electronic and thermal energies; H, Sum of electronic and thermal enthalpies; G, Sum of electronic and thermal free energies*.

In order to investigate the effect of the alkoxy chain length on the stability of the stable conformer **I/A***n* of the prepared SMHBCs, the thermal parameters have been calculated. The increment of the alkoxy chain length of the acid strongly enhances the calculated stability of the complexes. The long chain lengths increase the Van der Waal interaction between the alkoxy chains and consequently, decrease the predicted energy of SMHCs.

**Figure 6:**
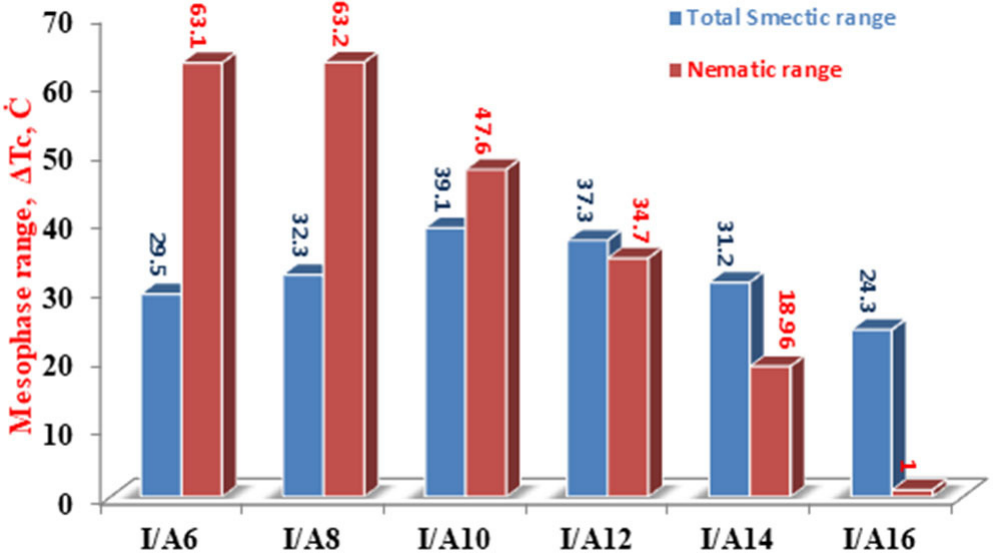
Dependence of the total (nematic and smectic) temperature ranges of **A***n* on the alkoxy-chain length.

**Figure 7:**
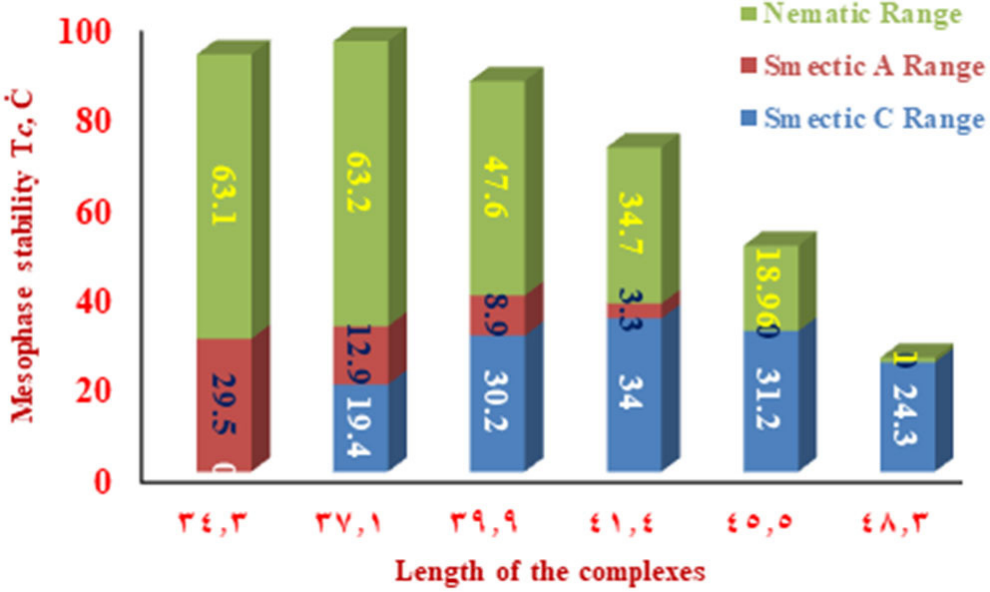
The dependence of the smectic A, C, and nematic temperature ranges on the molecular length of the SMHBCs **I/A***n*.

The change of the mesophases stability and their temperature ranges (**ΔT**) for the stable SMHBCs (**I/A***n*) with the alkoxy-chain length are shown in Table 3 and Figures 6, 7. It obvious from Figure 6 that the total smectic mesophase range is increased with the alkoxy chain length up to *n* =10 carbons then decreased again; however, the nematic mesophase range regularly decreases with the increase of the chain length. On the other hand, Figure 7 emphasizes that the nematic and smectic A stability of *H*-bonded complexes **I/A***n* decrease with the increase of the alkoxy chain length. As the alkoxy chain length increases, the length of the whole structure is increased successively, and consequently, the van der Waals intermolecular interaction increases. Such intermolecular interaction enhances the more ordered smectic C mesophase with the diminishing of the smectic A phase. Normally, the competitive lateral intermolecular and terminal interactions both affect the enhanced mesophase and their temperature ranges.

**Table 3:** Dipole moment (**μ**), polarizability (**α**), and aspect ratios (**L/D**) of SMHBCs, **I/A***n*.

**Parameters**		**I/A6**	**I/A8**	**I/A10**	**I/A12**	**I/A14**	**I/A16**
ΔT smectic C		0.0	19.4	30.2	34.0	31.2	24.3
ΔT smectic A		29.5	12.9	8.9	3.3	0.0	0.0
ΔT smectic (total)		29.5	32.3	39.1	37.3	31.2	24.3
ΔT nematic		63.1	63.2	47.6	34.7	18.96	1.0
ΔTc (total)		92.6	95.5	86.7	72.0	50.16	25.3
Tc (total)		196.7	195.9	178.4	165.6	147.16	125.5
μ Total		2.4002	1.1989	1.1506	1.1262	1.1140	1.1066
Polarizability α		590.73	635.36	680.72	724.29	768.66	590.73
Dimension Å	Width (D)	22.6	24.7	26.9	28.1	31.2	33.4
	Length (L)	34.3	37.1	39.9	41.4	45.5	48.3
Aspect ratio (L/D)		1.52	1.50	1.48	1.47	1.46	1.45

It is clear that the increase of the alkoxy chain length is accompanied by the decrease of the aspect ratio due to the bent shape of the SMHBCs, which are associated with increasing the width rather than the length. The negative effect observed on the total mesophase stability and its temperature ranges of all the *H*-bonded complexes **I/A***n* investigated can be accounted for in terms of the lower molecular packing. The longer chain length resulted in the dilution of the aromatic part of the diester base and consequently, decreases the aromatic packing. Such a decrease of the packing enhances the more ordered mesophase (smectic phase) within a lower temperature range. However, a decrease in the nematic thermal stability can be rationalized in terms of the effect aggregation of the terminal chains. The increment of chain length increases the strength of the terminal aggregation by increasing the van der Waals intermolecular interaction of the longer alkoxy chains with the enhancement of the total thermodynamic energy that results in the decrease of the nematic phase stability, Figure 7.

Generally, the dipole moment is considered to be one of the most important factors that affects the type and behavior of the formed mesophase. The lower the dipole moment of the *H*-bonded complexes may be safely attributed to the structural point of view. As the chain length increases the angle between the wings of the *H*-bonded complexes decreases (angle = 76° for **I/A***6* and 73° for **I/A***16*). Such small structural changes slightly affect the magnitude and the direction of the dipole moment. The variation of the dipole moment with the alkoxy chain could be an explanation for the formation of the nematic mesophase at the shorter chain lengths. The lower dipole moment value permits the terminal aggregation rather than the lateral one, which enhances the nematic mesophase; however, as the length increases the strong van der Waals interaction of the alkoxy chain enforces the parallel interaction which facilitates the formation of the smectic mesophase while encourages the shrinking of the less ordered nematic one.

**Figure 8:**
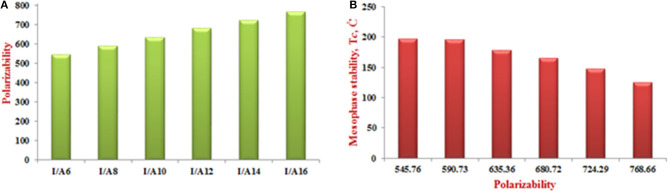
The dependence of the polarizability on the length of the alkoxy chains **(A)** and **(B)** transition stability on the polarizability of the complexes **I/A***n*.

As shown from Figure 8A, the predicted polarizability of the *H*-bonded supramolecular complexes **I*/*A***n* is a significant effect of the length of the alkoxy chain attached to the acid component. Increasing the alkoxy chain length by two carbons could enhance the polarizability successfully by 45 Bohr^3^. The increment of the alkoxy chains resulted in higher space filling and so increases the polarizability. On the other hand, the dependence of the mesophase thermal stability on the polarizability is shown in Figure 8B. As the polarizability increases, the mesophase stability increases accordingly. It is obvious, that the *H*-bonding complexation could improve the characteristics of the individual components, by changing the parameters that are needed for special applications, such as, the more polarizable compound possesses the essential characteristics of the liquid crystalline material to be suitable for electro-optical technology (Meredith et al., 1982; Khoo and Wu, 1993; Chemla, 2012).

### Frontier Molecular Orbitals and Polarizability

**Figure 9:**
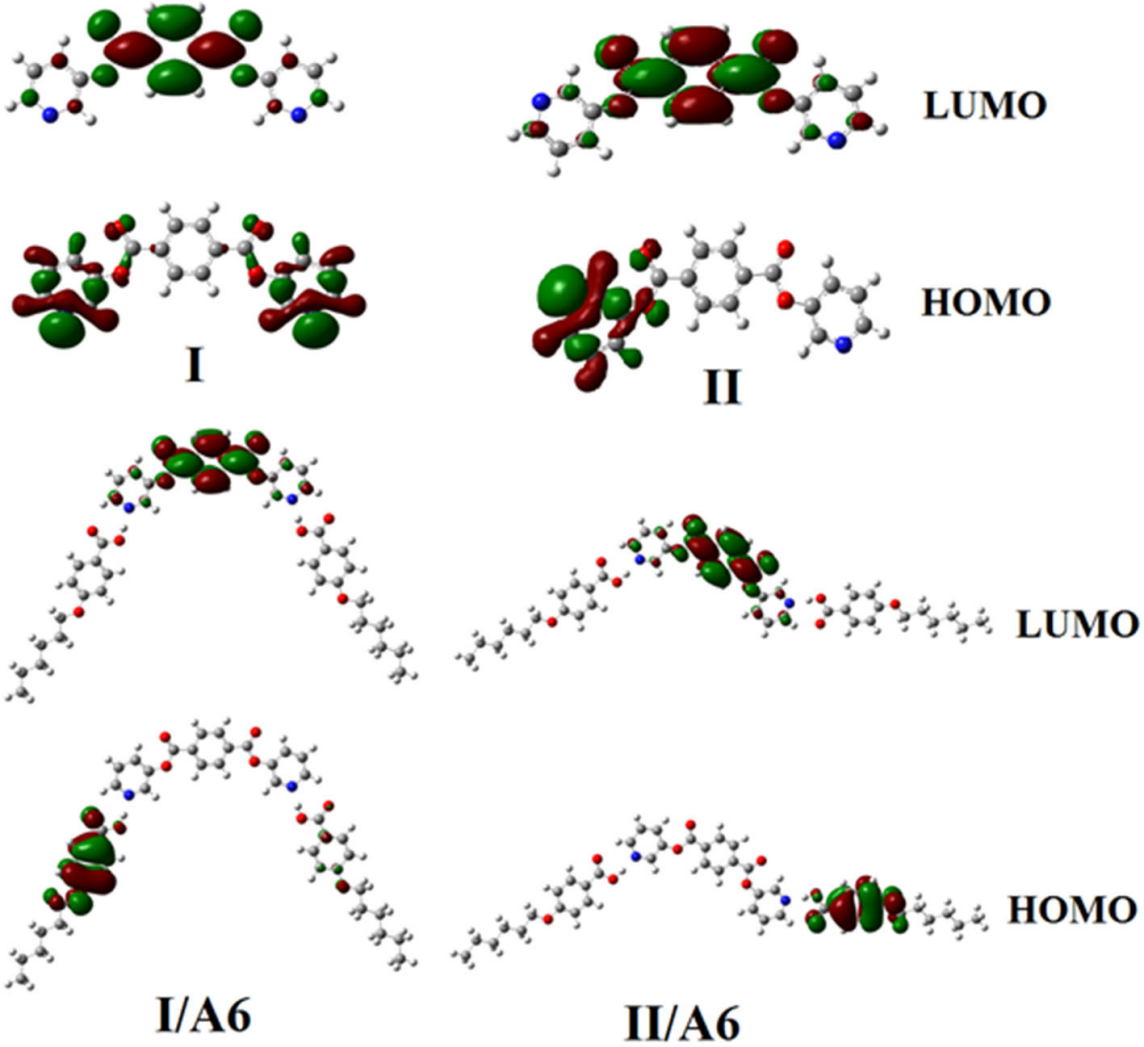
Estimated plots for frontier molecular orbitals of both conformers of SMHBCs, **I/A***n*, and **II/A***6*.

**Figure 10:**
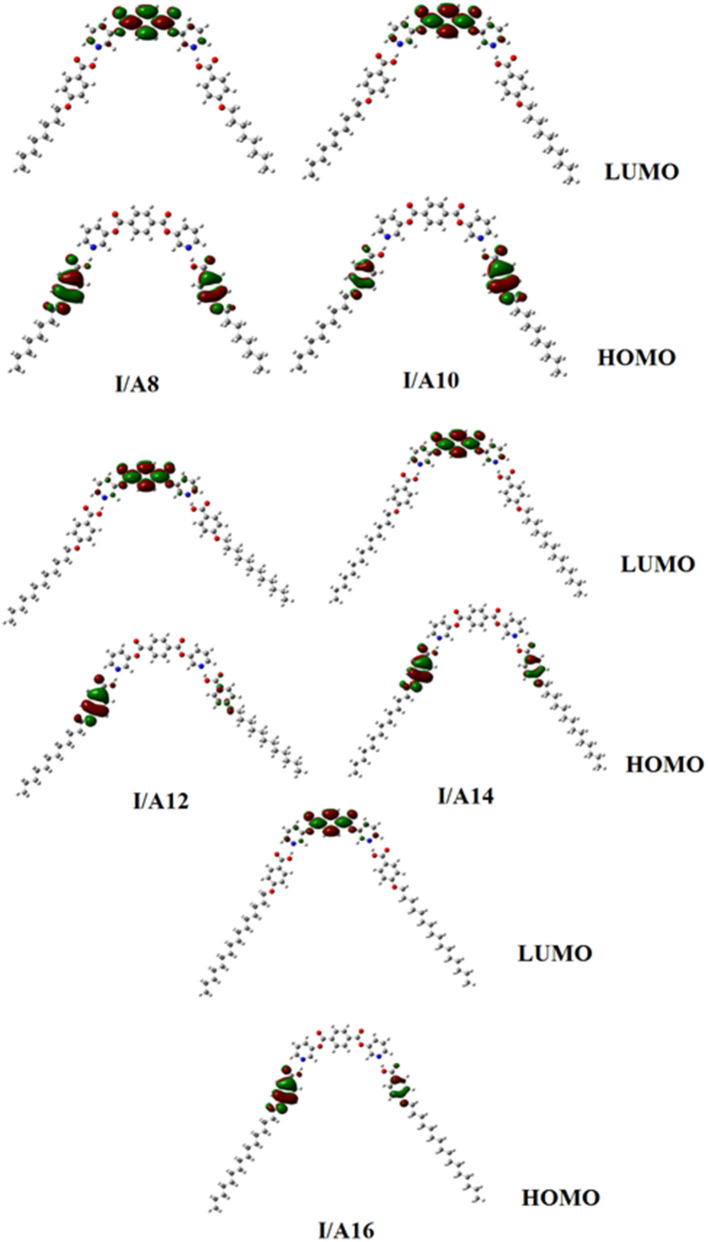
Estimated plots for frontier molecular orbitals of SMHBCs, **I/A***n*.

Figures 9, 10 demonstrate the estimated plots of frontier molecular orbitals (FMOs) HOMO (highest occupied) and LUMO (lowest unoccupied) for both conformers of the di-nicotinate base and their prepared **I/A***n* and **II/A6** complexes. It is obvious from the figure that the electron densities on the sites that are shared in the formation of the HOMOs of both conformers of base, **I** and **II**, were mainly localized on the nicotinate ring, and is shifted to the hydroquinone group for the LUMOs. On the other hand, the prepared SMHBCs showed that they shared the phenyl ring of the alkoxy acid in the formation of their HOMOs with the predominance of the di-nicotinate base in the formation of their LUMOs. Actually, there is no obvious impact of the direction of the *N*-atom or the alkoxy chain on the location of the electron densities of the FMOs. The energy difference between the FMOs could be used in the prediction of the ability of electron transformation from HOMO to LUMO during any electronic excitation process. The global softness (**S**) = **1/ΔE** is the parameter that inspects the polarizability and the sensitivity of materials for the photo-electric property. As shown from Table 4, the *H*-bonded complexes **I/A***6* and **II/A***6* derived from the di-nicotinate base **I** and **II** are in almost the same energy gap.

**Table 4:** FMO Energies a.u., polarizability, α, and dipole moment μ (Debye) of conformers of the di-nicotinate base and their SMHBCs, **I/An** and **II/A6**.

**Parameters**	**I**	**II**	**I/A6**	**I/A8**	**I/A10**	**I/A12**	**I/A14**	**I/A16**	**II/A6**
E_LUMO_	−0.1011	−0.1006	−0.1102	−0.1102	−0.1102	−0.1097	−0.1102	−0.1102	−0.1099
E_HOMO_	−0.2568	−0.2532	−0.2191	−0.2190	−0.2189	−0.2196	−0.2189	−0.2189	−0.2178
ΔE_HOMO−LUMO_	0.1556	0.1526	0.1089	0.1088	0.1088	0.1099	0.1087	0.1087	0.1080
S Softness	6.4251	6.5548	9.1802	9.1895	9.1946	9.1025	9.1996	9.2005	9.2618

### Molecular Electrostatic Potential (MEP)

**Figure 11:**
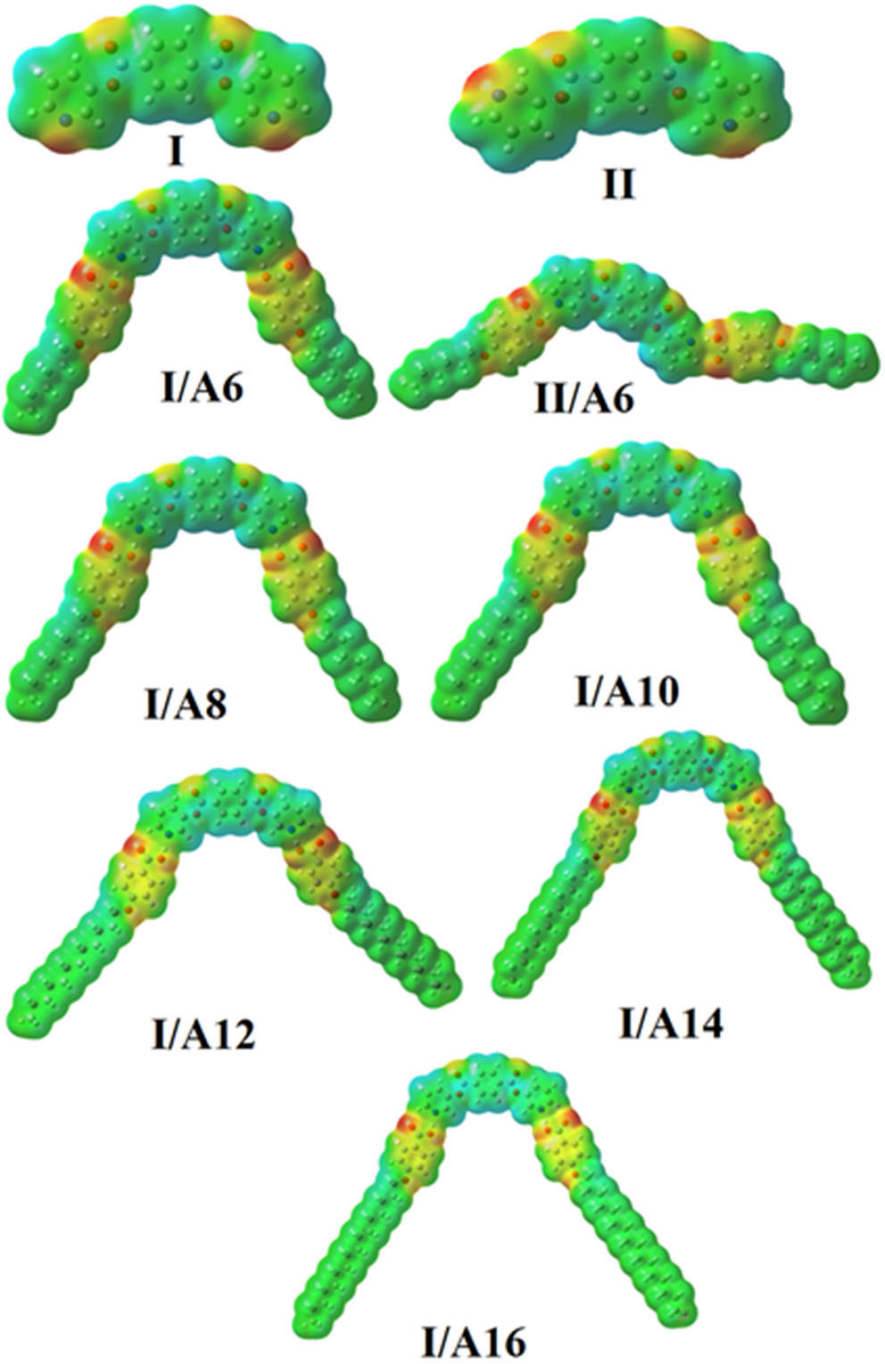
Molecular electrostatic potentials (MEP) for investigated complexes.

The mapping of the charge distribution (MEP) for SMHBCs of both the expected conformational isomers of the di-nicotinate base **I, II**, and their SMHBCs **I/A***n* and **II/A***6* were simulated applying the same method and at the same basis sets (Figure 11). The red region is considered the negatively charged atomic center and was estimated to be localized on the ester carboxylate linkage of the base **I** and **II**. However, for their complexes **I/A***n* and **II/A***6* the red region is localized on the H-bonded carboxylate moiety of the alkoxy acid. On the other hand, the part of the base as well as the electron donor alkoxy chains were predicted as the blue regions to show the lowest negatively charged atomic sites. As can be shown from Figure 11, the direction of the nitrogen atoms highly affects the degree as well as the orientation of the distribution of the charge mapping, **I/A***6* and **II/A***6*. Neither the orientation nor the amount of the charge distribution on the mapping are affected by length of the alkoxy chain of the SMHBCs **I/A***n*.

### Exchange of the Connecting Meso Core Effect

**Figure 12:**
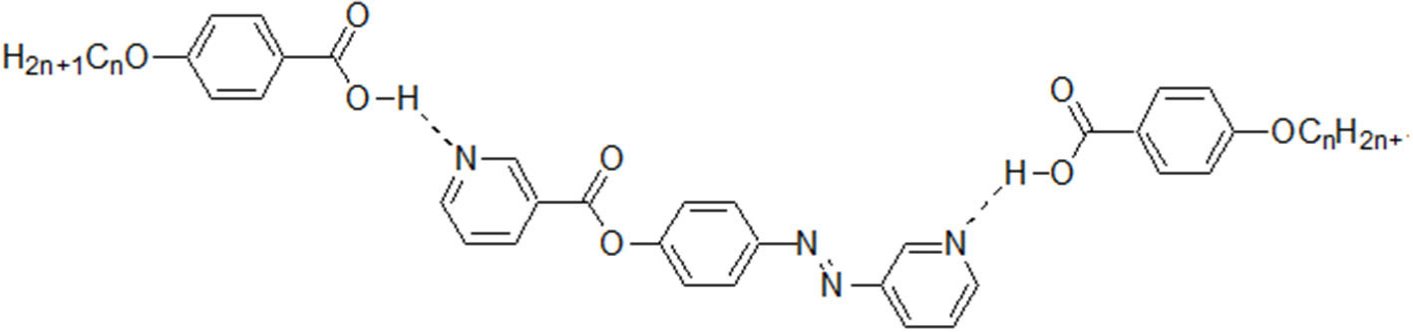
Our previously reported di-nitrogen SMHBCs analogs **III/A***n*.

**Figure 13:**
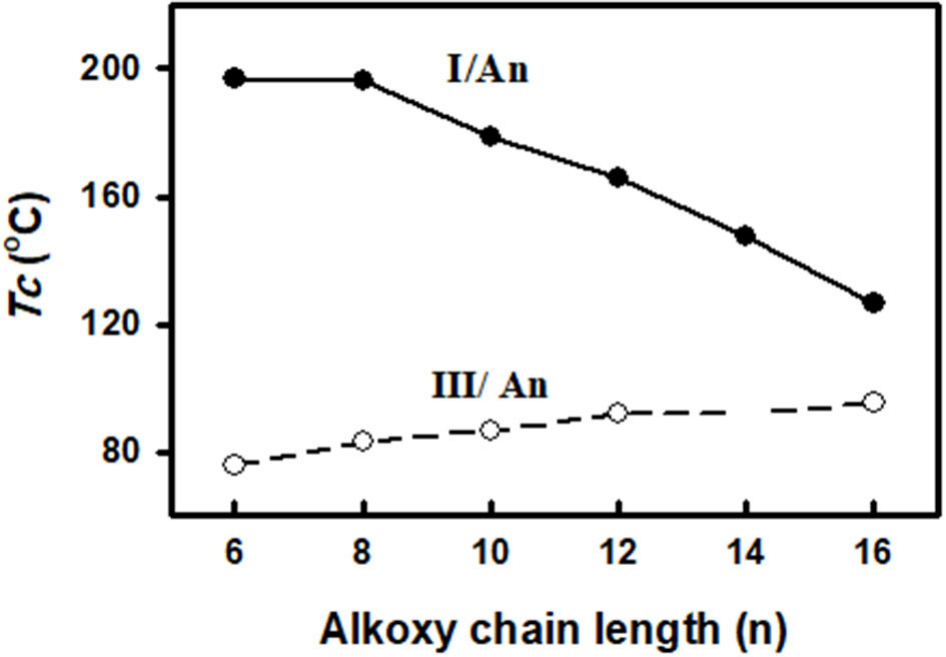
Comparison of the mesophase stability between the present SMHBCs **I/A***n* (•) and the previous system **III/A***n* (◦).

In order to study the effect of the exchange of one of the –COO– connecting units in the present diester derivatives with our previously reported (Nafee et al., 2020) SMHBCs of azo analog (-N=N–) in the basic moiety (**III/A***n*, Figure 12) on the mesomorphic properties, a comparison was established between mesophase stability (***T***_**C**_) of present investigated complexes **I/A***n* and the our previously reported five rings SMHBCs system data **III/A**n, Figure 13 and Table 5.

**Table 5:** FMO energies a.u., polarizability, α, and dipole moment μ (Debye) of SMHBCs, **(I, III)/A***8*.

**Parameters**		**-COO- di-linkages**	**-N=N- and –COO- linkages**
		**I/A*8***	**III/A*8***
E_LUMO_		−0.1102	−0.11849
E_HOMO_		−0.2190	−0.22681
ΔE_HOMO−LUMO_		0.1088	0.10832
S Global softness		9.18	9.23
μ Total		1.1989	4.6570
Polarizability α		635.4	656.72
Tc Smectic total		130.8	102.5
Tc total		195.9	110.6
ΔT smectic (total)		32.3	39.1
*ΔS/R*		2.4	7.87
Dimension Å	Width (D)	24.7	17.4
	Length (L)	37.1	51.5
Aspect ratio (L/D)		1.50	3.0

As can be seen from Figure 13 and Table 5, the replacement of the azo mesogenic group of the base **III** by the ester moiety in the di-nitrogen base resulted in a great enhancement of the thermal stability and induced a broad smectic phase covering all the chain lengths of alkoxy acid component. It is well known that the mesophase behavior of a certain liquid crystal molecule depends mainly on its mesomeric properties, namely inter molecular interactions, and the stereo conformation of the molecule. In the present supramolecular hydrogen-bonded complexes, **I/A***n*, the association of angular molecules, and consequently their mesophases stabilities, depend mainly on several factors:

1. Lateral adhesion of molecules which increases with the increase of either the alkoxy-chain length (*n*) or aspect ratio. The alkoxy chain length seems to play a significant role in the stabilization of mesophases of the SMHBC.
2. Molecular geometry which is actually a function of the structure of the complexes which in many cases is affected by the steric hindrance of the attached groups.
3. End-to-end interaction which depends mainly on the polarity and the length of the terminal substituents that result in the variation of the polarizability.

On the other side, the ester linking groups greatly affect the conjugation within the mesogenic core of the molecule.

Comparing the thermal stabilities of homologs of the prepared SMHBCs (**I/A***n*), revealed that each polarity, polarizability, aspect ratio, or planarity do not have similar effects on the mesophase behavior in our investigated complexes.

DFT results of thermal parameters and dimensional values revealed that the polarizability, the dipole moment, and the aspect ratio of the SMHBCs of azo derivative **III** is higher than that of the diester one **I**. All these factors could be evidence for the enhanced smectic mesophase range. The high aspect ratio and dipole moment of the SMHBCs of the azo derivative **III** enforces the lateral intermolecular interactions that permit the formation of the more ordered smectic mesophases. On the other hand, the lower values of these parameters for the diester SMHBCs of **I** facilitate the formation of the nematic mesophase in addition to the smectic ones.

On the other hand, although both SMHBCs are angular but the more linearity of the complexes **III/A***n* than **I/A***n* also resulted in the formation of the nematic mesophase of the complex **I/A***n* independent of length of the alkoxy chain. Such geometry permits longer terminal alkoxy chains aggregations to exhibit the nematic phase rather than the other homologs of higher dipole moment.

## Conclusions

A new type of SMHBCs complexes were reported and investigated experimentally and theoretically. Di-nicotinate (**I**) was taken as the proton-acceptor while the proton-donor was 4-n-alkoxybenzoic acid (**A***n*). Fermi-bands observed by FT-IR spectroscopy were attributed to the intermolecular *H*-bonding interactions. Mesomorphic properties of the prepared SMHBCs were thermally characterized by DSC and phases were identified by POM. Computational studies were established using DFT calculations. The results revealed:

1. All the prepared SMHBCs exhibited enantiotropic mesophases with wide ranges of thermal stabilities.
2. The complexes proved to be di or tri-morphic depending on the length of the terminal alkoxy chain.
3. The short terminal alkoxy chain complex (**I/A***6*) possessed SmA and N phases while the longer ones (**I/A***14* and **I/A***16*) exhibited both the SmC and N mesophases.
4. SMHBCs **I/A***8*, **I/A***10*, and **I/A***12* exhibited tri-mesophases (SmA, SmC, and N phases).
5. DFT calculations were based on two conformers of the di-nicotinate base and their prepared *H*-bonded complexes (**I/A***n* and **II/A***6***)**
6. The nematic and smectic A stability of the stable *H*-bonded complexes **I/A***6* decreased when increasing the alkoxy-chain length.
7. The effect of the replacement of one –COO– connecting unit with an azo group (-N=N–) in the basic moiety on the mesomorphic properties was investigated.
8. The DFT results revealed that the polarizability, the dipole moment, and the aspect ratio of the SMHBCs of azo derivative **III** was higher than that of the diester **I** and this could be the reason for the induced nematic mesophase of its complexes with 4-alkoxybenzoic acids**, I/A***n*.

## Data Availability Statement

The raw data supporting the conclusions of this article will be made available by the authors, without undue reservation.

## Author Contributions

LA-M, HA, and MH: data curation. LA-M, LA, and MH: formal analysis. LA-M, LA, and HA: funding acquisition. LA-M, LA, HA, and MH: methodology. MH: project administration and software. LA and HA: resources. HA, MN, and MH: writing—original draft, writing—review, and editing. All authors: contributed to the article and approved the submitted version.

## Conflict of Interest

The authors declare that the research was conducted in the absence of any commercial or financial relationships that could be construed as a potential conflict of interest.
